# Integrated Approach of Agri-nanotechnology: Challenges and Future Trends

**DOI:** 10.3389/fpls.2017.00471

**Published:** 2017-04-04

**Authors:** Sandhya Mishra, Chetan Keswani, P. C. Abhilash, Leonardo F. Fraceto, Harikesh Bahadur Singh

**Affiliations:** ^1^Department of Mycology and Plant Pathology, Institute of Agricultural Sciences, Banaras Hindu UniversityVaranasi, India; ^2^Institute of Environment and Sustainable Development, Banaras Hindu UniversityVaranasi, India; ^3^Laboratory of Environmental Nanotechnology, Institute of Science and Technology of Sorocaba, São Paulo State UniversitySão Paulo, Brazil

**Keywords:** agriculture, nanotechnology, biosynthesized nanoparticles, toxicity, bioavailability, sustainability, phytopathogens, soil

## Abstract

Nanotechnology representing a new frontier in modern agriculture is anticipated to become a major thrust in near future by offering potential applications. This integrating approach, i.e., agri-nanotechnology has great potential to cope with global challenges of food production/security, sustainability and climate change. However, despite the potential benefits of nanotechnology in agriculture so far, their relevance has not reached up to the field conditions. The elevating concerns about fate, transport, bioavailability, nanoparticles toxicity and inappropriateness of regulatory framework limit the complete acceptance and inclination to adopt nanotechnologies in agricultural sector. Moreover, the current research trends lack realistic approach that fail to attain comprehensive knowledge of risk assessment factors and further toxicity of nanoparticles toward agroecosystem components *viz*. plant, soil, soil microbiomes after their release into the environment. Hence in the present review we attempt to suggest certain key points to be addressed in the current and future agri-nanotechnology researches on the basis of recognized knowledge gaps with strong recommendation of incorporating biosynthesized nanoparticles to carry out analogous functions. In this perspective, the major points are as follows: (i) Mitigating risk assessment factors (responsible for fate, transport, behavior, bioavailability and toxicity) for alleviating the subsequent toxicity of nanoparticles. (ii) Optimizing permissible level of nanoparticles dose within the safety limits by performing dose dependent studies. (iii) Adopting realistic approach by designing the experiments in natural habitat and avoiding *in vitro* assays for accurate interpretation. (iv) Most importantly, translating environmental friendly and non-toxic biosynthesized nanoparticles from laboratory to field conditions for agricultural benefits.

## Introduction

The development of nanotechnology has provided an exciting and novel frontier to nearly all fields of industrial applications with profound impact on human’s life (Linkov et al., 2011). This key enabling technology has evidenced broad and remarkable applications in diverse fields such as electronics, medicines, cosmetics, textiles, food science, energy sector and agriculture (Caruthers et al., 2007; Sharon et al., 2010; Sastry et al., 2011; NCPI, 2011). The worldwide popularity and expansion of nanotechnology industry could be anticipated by the fact that its market value will reach to US$ 75.8 Billion by 2020 due to its significant expansion at global level (Research and Markets, 2015). Indeed, the waves of nanotechnology-based researches have expeditiously contributed to global growth by delivering strong applications in many aforementioned industrial sectors. Simultaneously, it is a well-known fact that nanotechnology has tremendous potential to benefit society by revolutionizing the agricultural sector. Basically, this innovative technology has subsidized the agricultural based business sector with annual growth rate of 25% (US$ 1.08 billion). Moreover, it is estimated that integration of advanced nanotechnology in agriculture would thrust the global economic growth to ∼ US$ 3.4 trillion by 2020 (Sodano and Verneau, 2014; Sabourin and Ayande, 2015). This clearly accentuates the relevance of agri-nanotechnological researches equipped with comprehensive knowledge on its environmental impact, biosafety concerns and regulatory issues.

**FIGURE 1:**
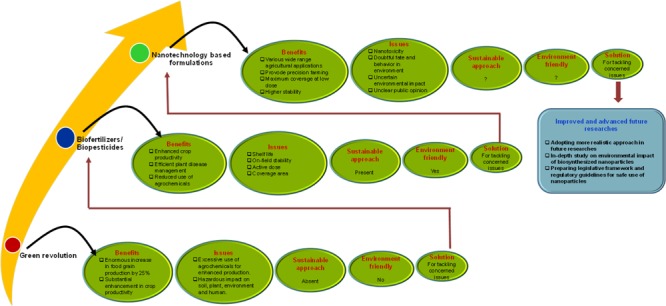
**An overview of breakthrough revolutions in the field of agriculture with their respective benefits, issues and solutions.** The schematic addresses the significance and superiority of nano-revolution over other two revolutions in agricultural sector.

Nanotechnology exhibiting multidisciplinary applications is recognized as sixth most revolutionary technology in the modern era (Knell, 2010). Among the preceding revolutions introduced at different timescale, the Green revolution of 1960s and currently nanotechnology have immensely affected the agricultural field (NAAS, 2013). The Green revolution has confronted major drawbacks associated with productivity, constancy, sustainability and equity leading to urgent need of novel concepts for agricultural research and progress (Conway and Barbie, 1988). The increased dependency on chemical pesticides and fertilizers during and post Green revolution has generated serious issues related to sustainability, environmental impact and health hazards. As a result, the innovative approach of using environment friendly biofertilizers/biopesticides as alternative to agro-chemicals came in existence to ensure biosafety (Mishra et al., 2015). However, this exciting approach also comprised of some major issues of poor shelf life, their on-field stability, performance under fluctuating environmental conditions and most importantly the high required dose for maximum coverage area. Interestingly, nanoparticles based formulations have shown superiority over bioformulations in terms of confronting all these issues (Navrotsky, 2000; Auffan et al., 2009). As a result, contemporarily, the modern agriculture is embracing the innovative approach of nanotechnology to combat global challenges of crop production, food security, sustainability and climate change (Nair et al., 2010; Ghormade et al., 2011; Mishra et al., 2014a) (**Figure 1**). In addition to agriculture, it is important to consider that nanotechnological applications have also proved its relevance in all areas of food science including food processing, food safety through improved packaging, enhancing food nutrition and superior quality food contact materials (Amenta et al., 2015; Handford et al., 2015). However, the underexplored areas of this important aspect leading to apparent impediments, negative perceptions and hesitant adoption of nanotechnology cannot be overlooked. In this context, the present article discusses the key knowledge gaps and further highlights the promising approaches for future agri-nanotechnology researches. Moreover, considering the growing concern about nanotoxicity, we urge to recommend the agricultural application of biosynthesized nanoparticles to enhance agricultural sustainability.

## Multifarious Applications of Nanotechnology in Agriculture and Identified Knowledge Gaps: An Overview

The potential benefits of nanotechnology in agricultural sector have created a great interest, as it can enhance agricultural productivity with low input of cost and energy. Importantly, nanotechnology by virtue of nanoparticles, has offered enormous potential applications in agriculture sector that include nanofertilizer, nanopesticide, nanoherbicide, nanosensor, and smart delivery systems for controlled release of agrochemicals (Salamanca-Buentello et al., 2005; Oliveira et al., 2014; Campos et al., 2014; Grillo et al., 2016). Additionally, nanotechnology based devices are also used for plant breeding and genetic engineering purposes (Jiang et al., 2013). The encouraging development of nanotechnological approaches in agriculture particularly for crop productivity and disease management is shown by current trends of publications and patents (Ghormade et al., 2011; Kah and Hofmann, 2014; Mishra et al., 2014b; Parisi et al., 2015; Mishra and Singh, 2016). To date, several studies have addressed how nanotechnological approach is benefiting the agricultural sector in number of ways. For examples: pesticides encapsulation in nanoparticles for their sustained release; nanoparticles mediated delivery of genetic material for crop improvement; carbon nanotube assisted seed germination of rain-fed crops; nanofertilizer for enhanced crop nutrition & crop productivity; nanopesticide for plant disease management; nanoherbicide for weed elimination and nanosensors for detection and forecast of pathogens and soil monitoring (Li et al., 2007; Barik et al., 2008; Wilson et al., 2008; Gan et al., 2010; Nair et al., 2010; Ghormade et al., 2011; Grillo et al., 2014; Mishra et al., 2014b; Oliveira et al., 2014; Campos et al., 2015a,b; Liu and Lal, 2015; Oliveira H. C et al., 2015; Oliveira J. L et al., 2015; Maruyama et al., 2016). Besides these, the probable impact of nanomaterials exposure on fate and accumulation of other organic and inorganic co-contaminants (being added to agricultural system such as pesticides, fertilizers, heavy metals etc.) is the recent development (Servin and White, 2016). Earlier report by De La Torre-Roche et al. (2013) suggest reduced uptake of pesticides by plants due to their association with carbon nanomaterials. This finding indicates beneficial impact of such interactions on reducing the pesticides residues in plants and edible parts. Given the importance to this aspect, such kind of studies must be promoted to remove the agricultural contaminants such as heavy metals and agrochemicals.

Undoubtedly, nanotechnology provides the possibility of precision farming (i.e., augmenting agricultural production with minimum input) in the era where elevating demand of sustainability compels to reduce the cost and excessive use of agricultural and natural resources (Chen and Yada, 2011). However, despite the exciting results obtained by involvement of ground-breaking nanotechnology in agriculture so far, their relevance have not yet reached up to the market. This is mainly attributed to small scale bench-top researches, ambiguous technical benefits, insufficient economic interest, biosafety concerns, regulatory issues and public opinion (Parisi et al., 2015). Additionally, the hovering apprehensions about fate, transport, bioavailability and toxicity of nanoparticles, limit the complete acceptance and willingness to adopt nanotechnology in agricultural sector. Nevertheless, nanotechnology renders precise capability to revolutionize the agricultural sector but at the same time it is also important to note that its concrete contribution to agriculture is still uncertain and at its nascent stage. Therefore, we need to include a system level approach providing more accurate informations on nanoparticles exposure and their risk in agricultural systems. In response to this, we highlight the future directions for improved agri-nanotechnology researches with special emphasis on; (i) optimization of the safe use of nanoparticles at permissible level for agricultural benefits by modulating the fate, behavior, bioavailability and toxicity determining factors (ii) advancement in experimental design and, (iii) incorporation of biosynthesized nanoparticles and assessing their relative advantages over nanoparticles from non-biological sources.

## Concerning Risk Assessment Factors and Their Modulation for Safe Use of Nanomaterials in Agriculture

Undoubtedly, the current scenario is witnessing the successful advancement and remodeling of agricultural sector due to captivating scientific applications of nanotechnology. Inevitably, such advancement is urgently required to boost the agricultural production in order to feed growing global population, which is likely to reach 9 billion by 2050 (Chen and Yada, 2011). Considering the expected benefits of nanotechnology-based products in agriculture in near future, several countries across the world are giving considerable efforts in evaluating the suitability and compatibility of integration of nanotechnology with agriculture. In this regard, agricultural scientists are trying to fill the knowledge gaps regarding toxicity of nanoparticles toward agro-ecosystem components mainly plant, soil and soil biota. As soon as nanoparticles are released in the environment, they are subjected to the possible interactions with these agro-ecosystem components (Anjum et al., 2013; Mishra and Singh, 2015a). Therefore, researchers are taking efforts to understand and scrutinize the extent of these major interactions in order to gain functional knowledge about toxicity and probable impact of released nanoparticles on environment and agriculture. Moreover, such investigations would primarily contribute to determine the permissible level of nanoparticles within tolerable safety limits.

The plant-soil interaction is the main driving force for agricultural production, which is influenced by any alteration in physico-chemical properties of soil system. Notably, soil is actually the paramount sink of released nanoparticles and hence, their subsequent interaction with different soil components could have profound impact on the fate, transport and behavior of nanoparticles. For instance, few reports on the most popular and most studied silver nanoparticles (AgNPs) possessing antimicrobial property have clearly indicated the importance of soil pH, organic matter content and cation exchange capacity in controlling their fate, toxicity and bioavailability (Jacobson et al., 2005; Shoults-Wilson et al., 2011; Benoit et al., 2013). It has been observed that lower range of soil pH, organic matter content and cation exchange capacity obstructs sorption of Ag to soil resulting into enhanced risk of mobility, toxicity and bioavailability of AgNPs. In contrary, higher range of soil pH, organic matter content and cation exchange capacity facilitates Ag sorption to soil that prevent mobility, bioavailability and further toxicity of AgNPs (Shoults-Wilson et al., 2011; Benoit et al., 2013; Mishra and Singh, 2015a; Klitzke et al., 2015; Schlich and Hund-Rinke, 2015). Likewise, Wang P. et al. (2013) made comparative analysis of ZnO-NPs toxicity in solution culture and soil system. The authors advocated substantial reduction in the toxicity of ZnO-NPs in soil system. This is mainly attributed to a range of soil characteristics (pH and cation exchange capacity) which determine phytotoxicity of Zn in soil system while on other hand particle dissolution in solution culture caused more toxicity. Furthermore, loamy sand soil with pH 5.5 was reported to exhibit no phytotoxicity of ZnO-NPs at concentration of 2000 mg kg^-1^ toward *Cucumis sativus* (Kim et al., 2011) whereas loamy clay soil with pH 7.36 showed noticeable toxicity toward *Triticum aestivum* at concentration of 45.45 mg kg^-1^ (Du et al., 2011). Additionally, soil organic matter is considered as another important key factor influencing transport behavior of ZnO-NPs that eventually determines their further toxicity. Zhao et al. (2013) revealed positive effect of alginate in reducing the toxicity of ZnO-NPs toward *Zea mays*. The ZnO-NPs added to soil together with alginate at concentration of 400–800 mg kg^-1^ exhibited no reduction in plant biomass whereas significant reduction was observed without alginate. As evident from these findings that soil environment can assess the potential environmental risk of nanoparticles and therefore we should recommend soil ecotoxicity studies with nanoparticles to predict their long term maximum effects.

As noted above, nanoparticles introduced in the environment ultimately accumulate in the soil and their fate, transport and behavior is largely affected by soil characteristics. In addition to this, most of the exisiting literatures have also focused on determining the direct impact of released nanoparticles on soil microbial community structure (Hänsch and Emmerling, 2010; He et al., 2011; Simonin and Richaume, 2015). In this regard, initially, Ge et al. (2012) envisaged potential effect of TiO_2_ and ZnO-NPs on soil bacterial community in a dose dependent manner. Using DNA-based fingerprinting analysis, they observed reduced bacterial diversity with declining taxa of Rhizobiales, Bradyrhizobiaceae, and Bradyrhizobium (related to nitrogen fixation) in response to these nanoparticles treatment. However, some positive impact was also observed on Sphingomonadaceae and Streptomycetaceae. It is interesting to note here that TiO_2_ and ZnO-NPs have significantly altered the bacterial community structure with distinct impact on environmental processes. For instance, the declining taxa are closely associated with nitrogen fixation process whereas increasing taxa are likely to affect the decomposition process of organic pollutants and biopolymers. Further, Shahrokh et al. (2014) also revealed dose dependent effect of AgNPs on nitrate reductase activity of *Rhizobium* and *Azotobacter*, where low dose of AgNPs (0.2 ppm) facilitated nitrate reduction activity in *Azotobacter*. Based on findings of such studies it has been anticipated that the denitrifying bacterial community is assumed to be highly susceptible to nanoparticles toxicity (Throbäck et al., 2007; VandeVoort and Arai, 2012). Despite the clearly known impact of nanoparticles on soil microbial community, there exists a dearth of literature providing apparent connection between soil factors and toxic behavior of nanoparticles toward soil biota (Calder et al., 2012; Chunjaturas et al., 2014; Shah et al., 2014; Mishra and Singh, 2015a). In this context, Frenk et al. (2013) evidenced effect of copper oxide (CuO) and magnetite (Fe_3_O_4_) nanoparticles on soil bacterial community in two different soil types (sandy loam and sandy clay loam). Interestingly, more adverse effects of both nanoparticles were detected in sandy loam soil with CuO exhibiting relatively strong influence on community composition and bacterial activity. More specifically, Rhizobiales and Sphingobacteriaceae being the most targeted taxa, were negatively affected by CuO in sandy loam soil. However, limited effects were also noticed in sandy clay loam soil where 1% CuO decreased community composition and oxidative potential but on other side, Fe_3_O_4_ nanoparticles did not change the bacterial community structure. Based on this finding, it is worth mentioning here that occurrence of clay part in the soil actually contributed to diminished toxicity of nanoparticles (Schlich and Hund-Rinke, 2015). Most recently, Shen et al. (2015) demonstrated ecotoxicological effects of ZnO-NPs on soil microbes on the basis of parameters including ammonification, respiration, dehydrogenase activity and fluorescent diacetate hydrolase activity. The adverse effects of ZnO-NPs on soil microbes under microcosm set up were found to be more pronounced in acidic and neutral soil, while alkaline soil possessed relatively low toxicity. Similar to this, toxicity of TiO_2_ NPs was found to be mainly influenced by soil pH and organic matter as reported by Simonin et al. (2015). The authors noticed significant reduction on carbon mineralization (parameter to study microbial community) in soil with high pH and organic matter content. Evidently, these findings highlight the significance of identifying the major soil variables such as soil type, soil organic matter content and soil moisture while evaluating the toxicity of nanoparticles toward microbes in soil habitat. Most importantly, gaining precise knowledge on the probable impact of different soil properties on the toxicity behavior of nanomaterials would help in evading the release of nanoparticles in the soil environment favoring its toxicity. Moreover, adopting improved soil management practices such as mulching, enhancing soil organic matter content in order to reduce probable chances of nanoparticles toxicity would also be beneficial.

Phytotoxicity of nanoparticles is another prevalent topic of discussion as plants have plentiful opportunity to interact with nanoparticles due to large surface area of leaves and root system. In addition, risk of nanoparticles toxicity is higher in plants due to their miniscule size that can easily translocate within plant body. It is believed that nanoparticles enter within the plant body through surface adsorption or traversing through small openings of the plants (Dietz and Herth, 2011). The available literatures clearly point toward the all possible impact (positive, negative and neutral effects) of nanoparticles on plant system (Stampoulis et al., 2009; Khodakovskaya et al., 2009; Su et al., 2009; Lee et al., 2010; Cifuentes et al., 2010; Zhang et al., 2012). It is important to consider that phytotoxicity of nanoparticles is primarily dependent on their size and concentration. Accordingly, Ma et al. (2010) reviewed that nanoparticles of size less than 5 nm can easily be translocated through the cell wall pores while nanoparticles of size upto 20 nm moves through plasmodesmata. Moreover, small sized nanoparticles may cause phytotoxicity even at lower concentration owing to its easy uptake by the plants and their further translocation inside plant system (Rico et al., 2011; Wang J. et al., 2013). In case of the most common metallic nanoparticles i.e. AgNPs, nanoparticles size is considered to play a key role in their phytotoxicity behavior. It has been observed that small sized AgNPs in the range of 5-10 nm possess higher toxicity (Rico et al., 2011; Qian et al., 2013; Vannini et al., 2014).

Another study by Asli and Neumann (2009) reported mechanical mode of inhibition of TiO_2_-NPs (30 nm) treatment on hydraulic conductivity and transpiration rate of *Z. mays* grown in hydroponic solution, by decreasing cell wall pore size of root from 6.6 to 3 nm while, no significant inhibitory effect was observed in soil grown plants. In contrast, stimulating impact of TiO_2_-NPs (2.5 g L^-1^) application on fresh and dry weight of *Spinacia oleracea* has been noticed by Yang et al. (2007). In addition, this elevated response was also observed in case of chlorophyll, protein and total nitrogen content in leaves. The authors related the stimulating impact to improved nitrogen photoreduction where treatment of TiO_2_-NPs on exposure to sunlight enhanced reduction of N_2_ to NH_3_ in plants grown in nitrogen deficient solution. Similarly, Song et al. (2012) also reported positive impact on duckweed (*Lemna minor*) grown in culture media supplemented with TiO_2_-NPs at very low concentration of 0.5 g L^-1^ whereas, higher concentration caused significant damage to the plants. The ZnO-NPs being the most widely used metal oxide NPs, have also been reported to enhance the growth of mung (*Vigna radiata*) and chickpea (*Cicer arietinum*) grown on plant agar media at concentration of 20 mg L^-1^ and 1 mg L^-1^, respectively (Mahajan et al., 2011). Interestingly, Zhao et al. (2013) evidenced plant growth promoting effect of ZnO-NPs on *Cucumis sativus* grown in soil system at concentration of 400 and 800 mg kg^-1^. However, higher dose of ZnO-NPs beyond this concentration limit caused phytotoxic effects. Similar dose dependent effect has been observed in case of Cu-NPs that noticeably inhibited the growth of wheat (*T. aestivum*) and mung bean (*Phaseolus radiatus*) at concentration of 570 and 335 mg L^-1^ respectively. For homogenous exposure of nanoparticles suspension to the plants and to avert the possibility of precipitation, this test was conducted in plant agar media (Lee et al., 2008). Likewise, higher concentration of 1000 mg L^-1^ of Cu-NPs prominently reduced the growth of zucchini (*Cucurbita pepo* cv *costata romanesco*) grown in Hoagland solution (Stampoulis et al., 2009). On the contrary, it is worth mentioning here that stimulating impact was observed in lettuce (*Lactuca sativa*) plants grown in soil amended with 130 and 600 mg kg^-1^ of Cu-NPs (Shah and Belozerova, 2009). Typically, other type of nanomaterials, e.g., multi-walled carbon nanotubes (CNT) were also reported to enhance seed germination and root elongation of ryegrass at concentration of 2 g L^-1^, while no significant effects were noted on radish, lettuce, cucumber seeds (Lin and Xing, 2007). Likewise, application of 50 mg L^-1^ CNTs has also been indicated to enhance the yield of tomato by improving water use efficiency of plants (Khodakovskaya et al., 2013). Altogether, these studies strongly highlight the point that phytotoxic behavior of nanomaterials is largely dependent on concentration and plant growth system. Considering the relevance of nano-phytotoxicity in agro-ecosystem, it should be noted that soil is the main route through which plants are largely exposed to the released nanoparticles. Hence, pointing to this fact, more realistic approach must be incorporated in our experimental design to gain appropriate knowledge about the fate and risk of nanoparticles toxicity toward plants. Therefore, many researchers have started to consider this approach by avoiding hydroponics system so as to get more relevant toxicity data and unambiguous interpretation. With reference to this, previously, Zhu et al. (2008) interpreted nanofilter capability of soil system as it prevented uptake of Fe_3_O_4_-NPs by *Cucurbita maxima*, while 1.3% of 0.5 g L^-1^ Fe_3_O_4_-NPs (20 nm) was traced to translocate to the leaves when the plants were grown in hydroponics growth system. Likewise, CeO_2_ nanoparticles (37 nm) when applied to soil at 10 μg g^-1^ through 14 days irrigation, yielded neutral effect on maize plants because neither noticeable uptake nor any kind of growth inhibition was recorded (Birbaum et al., 2010). Additionally, Lee et al. (2012) also evidenced low toxicity of AgNPs toward *Phaseolus radiatus* and *Sorghum bicolor* grown in soil system as compared to agar. Most importantly, the response of plants to nanoparticles released in soil system is largely governed by soil parameters but unfortunately, there is lack of research on this aspect. However, only few studies have investigated this approach, for example, Jośko and Oleszczuk (2013) demonstrated non-toxic effect of ZnO-NPs toward *Lepidium sativum* grown in soil with higher level of cation exchange capacity. Likewise, Dimkpa et al. (2012) observed higher toxicity of CuO and ZnO nanoparticles toward *T. aestivum* grown in sandy soil. Based on these findings, there is clear evidence that soil parameters can modulate the probable phytotoxicity of nanoparticles. Therefore, considerable efforts must be undertaken in this direction for gaining detailed understanding of multitude of soil components affecting the plant-nanoparticles interaction in soil and for making reliable estimate of phyto-nanotoxicity.

In light of the above-mentioned reports, we assume that an understanding of the environmental impact of nanoparticles released in agro-ecosystem must include the analysis of basic risk assessment factors during the tripartite interactions of nanoparticles with plant, soil, and soil microbial community (**Table 1**). Indeed, the key soil factors mainly soil type, pH, organic matter content, soil moisture, govern the behavior, and toxicity of released nanoparticles toward plants and microbes. However, on other side, phytotoxicity of nanoparticles is largely regulated by their size, concentration and plant growth system. Here, the important point is to mitigate these risk assessment factors for alleviating the subsequent toxicity of nanoparticles.

**Table 1:** Review of the possible interactions and impact of nanomaterials on soil microbes and plant under varying soil physico-chemical properties.

Soil parameters	Nanomaterials	Major findings	References
**Soil types**			
Silty clay	TiO_2_	Significantly lowered carbon mineralization	Simonin et al., 2015
Sandy loam	TiO_2_	Adverse impact on soil microbial community	Simonin et al., 2015
	CuO, Fe_3_O_4_	Negative effect on soil microbial community	Frenk et al., 2013
	ZnO	No toxicity on *Cucumis sativus* with soil pH 5.5 and at concentration of 2000mg/kg	Kim et al., 2011
	CuO, ZnO	Toxic effect on *Triticum aestivum*	Dimkpa et al., 2013
	AgNPs	Reduced microbial biomass	Hänsch and Emmerling, 2010
		Reduced soil enzymatic activities and substrate induced respiration	Colman et al., 2013
	CeO_2_, Fe_3_O_4_, SnO_2_	No effect on microbial biomass C and N	Vittori Antisari et al., 2013
	TiO_2_	Reduced bacterial diversity	Ge et al., 2013
	TiO_2_ and ZnO	Reduced microbial biomass and substrate induced respiration	Ge et al., 2011
	TiO_2_, ZnO	Altered soil bacterial community with reduced taxa	Ge et al., 2012
Loamy clay	ZnO	Toxic effect on *Triticum aestivum* in soil pH 7.36 and at concentration of 45.45 mg/kg	Du et al., 2011
**pH**			
Acidic	AgNPs, ZnO	Enhanced toxicity toward *Eisenia fetida* Adverse effect on ammonification, respiration and dehydrogenase activity of soil microbes	Shoults-Wilson et al., 2011; Shen et al., 2015
Alkaline	TiO_2_	Significant reduction in soil microbial community	Simonin et al., 2015
	AgNPs	Declined toxicity toward soil microbial activity	Schlich and Hund-Rinke, 2015
**Organic matter**			
High	AgNPs	Reduced toxicity toward biofilm forming communities	Sheng and Liu, 2011; Wirth et al., 2012
	TiO_2_	Toxic effect on microbial activity, i.e., carbon mineralization	Simonin et al., 2015
	ZnO	Positive impact on *Zea mays* when alginate added at concentration of 400–800 mg/kg	Zhao et al., 2013
Low	CuO, Fe_3_O_4_	Enhanced toxicity toward microbial community	Frenk et al., 2013
**Cation exchange capacity**			
High	AgNPs	Reduced toxic impact on soil bacterium *Pseudomonas chlororaphis* O6	Calder et al., 2012
	ZnO	Non-toxic effect on *Lepidium sativum*	Jośko and Oleszczuk, 2013
Low	AgNPs	Enhanced toxicity toward soil microbes	Calder et al., 2012

## Comprehensive Understanding of the Interactions, Toxicity, and Fate of Biosynthesized Nanoparticles

The rapid development of nanotechnology has raised several issues of which synthesis protocols are gaining considerable attention. In general, a variety of physical and chemical protocols *viz*. laser pyrolysis or ablation, micro-emulsion sol-gel, ultrasonic fields, UV irradiation photochemical, reduction techniques etc. have been successfully employed for nanoparticles synthesis (Sastry et al., 2004; Yang and Aoki, 2005; Aslan et al., 2006; Cao and Hu, 2009). However, feasibility of these protocols is still ambiguous and contentious due to environmental risks of toxic and hazardous chemicals used for synthesis purpose (Li et al., 2011; Singh et al., 2016). Therefore, opting ecofriendly, non-toxic and sustainable methods for fabricating a myriad of nanoparticles is the current area of global interest. In this way, several biological agents such as bacteria, fungi, actinomycetes, plants and algae have been exploited for biosynthesis of nanoparticles (Ahmad et al., 2003; Singaravelu et al., 2007; Li et al., 2011; Mittal et al., 2013; Mishra et al., 2014b). The superiority of biological method for nanoparticles synthesis could be estimated by the fact that the whole process of synthesis is rapid, stable and requires a wide range of non-toxic biomolecules of low cost and most importantly provides more stable nanoparticles (Singh et al., 2016; Hussain et al., 2016). Moreover, shape and size of the nanoparticles can also be regulated by modifying the pH and temperature of the reaction mixture (Gericke and Pinches, 2006). Thus, several metal nanoparticles (Au, Ag, Fe, Pt, Ti, Zn, Mg etc.) have been successfully fabricated using biosynthetic approach (Kharissova et al., 2013). Interestingly, biosynthesized nanoparticles are found to show improved activity in comparison to those synthesized by physical and chemical methods (Sintubin et al., 2011). However, besides these advantages, the major concern associated with biological synthesis approach is the polydispersity of synthesized nanoparticles. But by optimizing the synthesizing conditions such as pH, temperature, salt concentration; the shape, size and dispersity of nanoparticles can be controlled (Pimprikar et al., 2009; Iravani et al., 2014).

In relation to agricultural perspective, biosynthesized nanoparticles offer efficient and environment-friendly applications particularly for plant growth promotion, plant disease management and stress tolerance (Mishra et al., 2016). In this context, Raliya et al. (2015) found stimulating impact of biosynthesized TiO_2_ nanoparticles using *Aspergillus flavus* on plant growth of *Vigna radiata* and rhizospheric microbial population. Likewise, Mishra et al. (2014b) reported strong antifungal activity of biosynthesized silver nanoparticles (AgNPs) against *Bipolaris sorokiniana*, spot blotch pathogen of wheat (*T. aestivum*). Additionally, biosynthesized AgNPs using *Serratia* sp. BHU-S4 were also found to inhibit melanin biosynthesis genes in *B. sorokiniana* (Mishra and Singh, 2015b). Apart from this, many earlier studies have confirmed the antimicrobial activity of biosynthesized AgNPs against broad range of phytopathogens pointing toward their exciting possibilities in agriculture (Krishnaraj et al., 2012; Gopinath and Velusamy, 2013; Lee et al., 2013; Paulkumar et al., 2014). Furthermore, Raliya et al. (2014) demonstrated positive effect of biosynthesized MgO nanoparticles using *Aspergillus flavus* on clusterbean (*Cyamopsis tetragonoloba*). Application of these nanoparticles at concentration of 15 mg L^-1^ resulted into enhanced root-shoot growth and chlorophyll pigment in clusterbean. Additionally, ZnO nanoparticles synthesized from *Aspergillus fumigatus*, showed significant improvement in overall plant health along with enhancement in rhizosphere microbial population, acid phosphatase, alkaline phosphatase and phytase activity in clusterbean rhizosphere (Raliya and Tarafdar, 2013). Correspondingly, Sabir et al. (2014) also proposed that application of ZnO-NPs can revolutionize the agricultural sector and could solve the current problem of food demand due to their antimicrobial and fertilizer action, especially if considered biogenic synthesis of these nanoparticles. The above-mentioned studies confirmed that agricultural applications of biosynthesized nanoparticles provide new insight on precision farming technology. Moreover, there is a growing interest in studying the fate, transport and toxicity of biosynthesized nanoparticles and hence more attention should be given in this direction.

Biosynthesis routes employ biological materials such as plant extracts, sugars, polyphenols, vitamins and microorganisms which are used as reducing and capping agents in synthesis process leading to more stabilized and biocompatible nanoparticles with higher longevity (Parsons et al., 2007; Kalaiarasi et al., 2010; Kharissova et al., 2013). Most importantly, the biofabricated nanoparticles exhibit relatively lower toxicity compared to chemically produced nanoparticles (Sanchez-Mendieta and Vilchis-Nestor, 2012; Varma, 2012; Órdenes-Aenishanslins et al., 2014). Consequently, with the growing public concern on the nanotoxicity and its direct or indirect environmental impact, considerable attention is required for employing biosynthesized nanoparticles for agricultural purposes. However, there is complete lack of studies aimed at toxicity, associated risk factors and environmental impact of biosynthesized nanoparticles. Furthermore, there is enormous scope of research in this underexplored, emerging and challenging area and hence, considerable efforts must be devoted to in-depth study on environmental impact of biosynthesized nanoparticles. Keeping this view in mind, we believe that meticulous application of biosynthesized nanoformulations in agricultural system would eventually remove its negative perception.

## Regulatory Policies and Considerations

Regardless of the promising development of nanotechnology in varied fields, its agricultural applications have not been translated to meet global needs primarily due to shallow awareness and biosafety concerns. The foremost reason for scarcity of commercial development of nanotechnology in agriculture is geographically limited existence of legislative framework, regulatory guidelines and negative public opinion (Arts et al., 2014). The growing challenge of global food security and climate change strongly underline the commercial applications of nanotechnology-based products for agricultural sector (Rossi et al., 2014). Therefore, there is an urgent need to thoroughly assess the risk assessment and risk management factors associated with application of nanoparticles in agricultural sector, prior to implementation of regulatory guidelines (FAO/WHO, 2013; Amenta et al., 2015). In this context, several regulatory bodies *viz*. US Food and Drug Administration (USFDA), Organisation for Economic Cooperation and Development (OECD), International Standard Organization (ISO) are undertaking challenges in this direction thereby agencies including USFDA specifically enforces legislation on soil while, ISO and OECD only provide guidelines and suggestions to the regulatory bodies. Different approaches are being followed in OECD and non-OECD countries in regulating nanotechnology in agri/feed/food sectors (Amenta et al., 2015). The main EU regulation is the REACH (Registration, Evaluation, Authorisation and Restriction of Chemicals) which chiefly addresses the use of nanomaterials in plant protection products, food additives/supplements and food contact materials (European Commission, 2013). Globally, only EU and Switzerland have successfully established nano-specific legislative provisions particularly for agriculture, food and feed sector whereas, other non-EU countries have non-mandatory frameworks binding with non-legal guidance (OECD, 2013). However, it is important to note that due to uncertainty of regulatory frameworks and difference in opinion across the globe, the nanoparticles based products for agricultural benefits are not flourishing and facing difficulties in reaching to the market. Risk assessment and risk management are the top most priorities to be considered in framing regulatory policies for addressing biosafety issues. Moreover, sharing views and opinion on public platform across the globe would be helpful in dealing with efficient regulatory measures.

## Recommendations for Future Research

**FIGURE 2:**
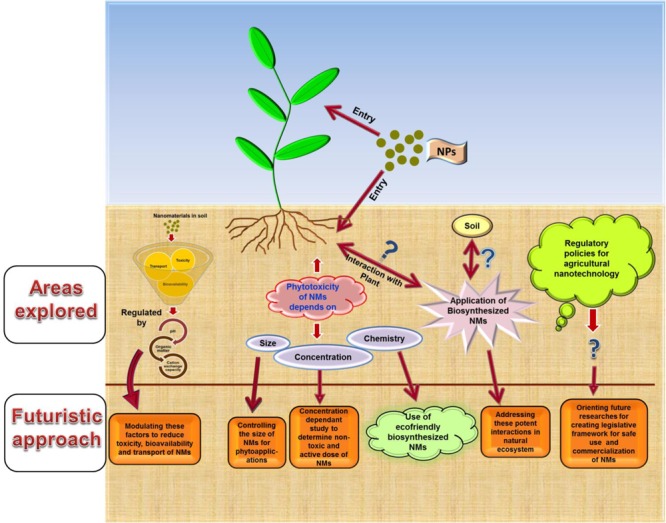
**Schematic representation of the key points to be addressed in future researches on agri-nanotechnology for filling in the identified knowledge gaps**.

Promising applications of nanotechnology in all areas cannot be overlooked. However, simultaneously uncertainty and negative perception *vis-à-vis* nanotechnological interventions in agricultural sector must be taken seriously. Hence, there is need to make extensive efforts in forwarding and improving the futuristic researches based on recognized knowledge gaps (**Figure 2**). In this context, we suggest the following key points:

✓ The future researches must be emphasized toward searching the ways to circumvent the risk factors associated with nanoparticles usage. Studying nanoparticles synthesis and bestowing few applications limited to laboratory conditions only could not contribute to the complete acceptance of nanotechnology in agricultural sector. Hence, scientific community must work together to improve the future researches based on more realistic approach.
✓ Validating the permissible level of nanoparticles dose within safety limits need to be explored and clarified. This could be achieved by attempting concentration dependent study in natural soil system in order to understand the accurate active and non-toxic dose of nanoparticles.
✓ An understanding of the transgenerational and trophic chain transfer effects of nanoparticles applications on plants must be included to gain comprehensive knowledge of nanotoxicity. Interestingly, selection of permissible level together with studying transgenerational and trophic chain transfer effects could provide adequate safety assessments.
✓ A clear overview of the soil physico-chemical characteristics of the agricultural fields where nanoparticles are to be applied may help in reducing their risk toward plant and soil biota. Altering soil environment in a way to modify the fate, transport and bioavailability of nanoparticles in order to reduce their subsequent toxicity could greatly achieve their safe and beneficial applications in agriculture. For example, advanced soil management practices for improving soil conditions could assist in reducing transport, bioavailability and further toxicity of nanoparticles with significant positive impact in agroecosystem.
✓ Finally, and most importantly, we strongly recommend the inclusion of biosynthesized nanoparticles as prerequisites for consequential and in-depth researches. Redeeming the environment-friendly approach of green synthesis of nanoparticles, it is believed that biosynthesized nanoparticles may possess relatively lesser or no toxicity and hence future researches must precisely focus on their practical utility. In addition, experimental design must be set in natural environment (growing the plants in soil) to give precise depiction of environmental impact of nanoparticles.

## Author Contributions

SM and HS conceived the idea of review, provided inputs for specific sections, and edited the final draft. The primary manuscript was written by SM and CK. PA and LF provided specific comments. All the authors read and approved it for publication.

## Conflict of Interest Statement

The authors declare that the research was conducted in the absence of any commercial or financial relationships that could be construed as a potential conflict of interest.
